# Genital Dysbiosis and Different Systemic Immune Responses Based on the Trimester of Pregnancy in SARS-CoV-2 Infection

**DOI:** 10.3390/ijms25084298

**Published:** 2024-04-12

**Authors:** Giuseppina Campisciano, Alice Sorz, Carolina Cason, Nunzia Zanotta, Fabrizia Gionechetti, Maria Piazza, Petra Carli, Francesca Maria Uliana, Lisa Ballaminut, Giuseppe Ricci, Francesco De Seta, Gianpaolo Maso, Manola Comar

**Affiliations:** 1 Department of Advanced Translational Microbiology, Institute for Maternal and Child Health—IRCCS Burlo Garofolo, Via dell’Istria 65, 34137 Trieste, Italy; carolina.cason@burlo.trieste.it (C.C.); nunzia.zanotta@burlo.trieste.it (N.Z.); petra.carli@burlo.trieste.it (P.C.); francescamaria.uliana@burlo.trieste.it (F.M.U.); lisa.ballaminut@burlo.trieste.it (L.B.); manola.comar@burlo.trieste.it (M.C.); 2 Department of Obstetrics and Gynecology, Institute for Maternal and Child Health–IRCCS Burlo Garofolo, Via dell’Istria 65, 34137 Trieste, Italy; alice.sorz@burlo.trieste.it (A.S.); maria.piazza@burlo.trieste.it (M.P.); giuseppe.ricci@burlo.trieste.it (G.R.); deseta.francesco@hsr.it (F.D.S.); gianpaolo.maso@burlo.trieste.it (G.M.); 3 Department of Life Sciences, University of Trieste, Via Licio Giorgieri 5, 34127 Trieste, Italy; fgionechetti@units.it; 4 Department of Medicine, Surgery and Health Sciences, University of Trieste, Strada di Fiume 447, 34149 Trieste, Italy; 5 Department of Obstetrics and Gynecology, IRCCS San Raffaele Scientific Institute, Vita-Salute San Raffaele, Via Olgettina 60, 20132 Milano, Italy

**Keywords:** COVID-19, pregnancy, dysbiosis, dysimmunity, SARS-CoV-2

## Abstract

Respiratory infections are common in pregnancy with conflicting evidence supporting their association with neonatal congenital anomalies, especially during the first trimester. We profiled cytokine and chemokine systemic responses in 242 pregnant women and their newborns after SARS-CoV-2 infection, acquired in different trimesters. Also, we tested transplacental IgG passage and maternal vaginal–rectal microbiomes. IgG transplacental passage was evident, especially with infection acquired in the first trimester. G-CSF concentration—involved in immune cell recruitment—decreased in infected women compared to uninfected ones: a beneficial event for the reduction of inflammation but detrimental to ability to fight infections at birth. The later the infection was acquired, the higher the systemic concentration of IL-8, IP-10, and MCP-1, associated with COVID-19 disease severity. All infected women showed dysbiosis of vaginal and rectal microbiomes, compared to uninfected ones. Two newborns tested positive for SARS-CoV-2 within the first 48 h of life. Notably, their mothers had acute infection at delivery. Although respiratory infections in pregnancy are reported to affect babies’ health, with SARS-CoV-2 acquired early during gestation this risk seems low because of the maternal immune response. The observed vaginal and rectal dysbiosis could be relevant for neonatal microbiome establishment, although in our series immediate neonatal outcomes were reassuring.

## 1. Introduction

Pregnancy renders women more susceptible to infections because of physiological changes, such as the weakening of the immune system and perturbation of the vaginal microbiome [1,2].

Infection with SARS-CoV-2 during pregnancy is rarely vertically transmitted to the fetus [3,4] and neonatal infection is also a rare event with the majority of infected newborns being asymptomatic [5] and usually not associated with adverse outcomes [6,7]. Anyway, the risk of adverse outcomes has been documented especially in women acquiring the infection during the third trimester of pregnancy [8,9,10,11].

Regardless of the low risk of vertical transmission and even in the absence of in utero infection, it is plausible that the consequences of the infection could impact the fetus, probably via the exacerbation of maternal immune activation, including inflammation [4,12,13]. Therefore, investigating the immune scenario acting in response to SARS-CoV-2 in both maternal and umbilical cord blood at delivery may shed light on the risk of sequelae. Similarly, for influenza A virus infection during pregnancy, mice models have shown that virus dissemination to maternal blood causes a “vascular storm”, characterized by a strong proinflammatory response and release of antiviral mediators, which could subsequently induce hypoxia in the placenta and fetal brain [14]. It is suggested that the timing of viral infection during fetal development may affect the duration of the immune response at the maternal–fetal interface and, thus, increase possible consequences for the fetus [15]. During the pandemic, information on whether the same risk may be associated with COVID-19—specifically, only acute or extended infection—was scarce or missing [11,16,17]. On the other hand, it seems that maternal antibodies against the virus persist throughout pregnancy, contributing to the protection of the newborn via transplacental transfer [18,19].

A neglected aspect in studies surveying SARS-CoV-2 infection during pregnancy is the description of the vaginal and gut microbiome of pregnant women, already known to influence the physiological colonization of the fetus and newborn. In the third trimester of pregnancy, the gut microbiome is populated by bacterial species implicated in energy production and storage to support the fetus’s growth [20] and, in the vagina, a shift toward a Lactobacillus-dominated microbiome has been described, playing a significant role in the reduction of the preterm and spontaneous labor [21,22].

Through a multidisciplinary approach, this study aims to investigate how infection of SARS-CoV-2 acquired at different gestational periods may behave differently. Nasal swabs of the pregnant women and of the newborn were tested for SARS-CoV-2 infection. At the time of delivery, viral infection was evaluated in the vagina, rectum, urine, and placenta. At the same time, IgG-specific immune response and a panel of cytokines, chemokines, and growth factors implicated in inflammation were measured in maternal sera and cord blood and vaginal and rectal microbiomes were additionally profiled. A group of pregnant uninfected women were investigated as a control group.

## 2. Results

### 2.1. Study Cohort

Two hundred forty-two pregnant women positive for SARS-CoV-2 were enrolled in the study and divided into three groups: (1) women positive during pregnancy (first or second trimesters) but negative at the time of delivery (Early-CoV group) (*n* = 176); (2) women positive at the time of delivery (Late-CoV group) (*n* = 58); (3) women positive at the time of delivery in nasal swabs and in one of the other biological samples tested (Spread-CoV group) (*n* = 8). In this last group, one woman showed SARS-CoV-2 in a vaginal swab, two women in rectal swabs, three women in placental swabs/placental biopsies, and the two remaining women showed SARS-CoV-2 in all the tested samples.

In terms of the delivery week, mode of delivery, maternal complications, and associated risk factors, there were no significant differences among the three groups. Symptomatology was mild in all the women except for six of them (two women from the Early-CoV group and four from the Late-CoV group), for whom COVID-19 pneumonia was diagnosed (6/242, 1.6%). Two newborns from mothers positive for SARS-CoV-2 (one from the Late-CoV group and one from the Spread-CoV group) were found positive at the time of delivery (2/242, 0.82%) (Table 1).

### 2.2. SARS-CoV-2 Antibody Transplacental Passage

SARS-CoV-2-specific IgG was tested in maternal serum and cord blood sample. Regarding the maternal blood, in the Early-CoV group (*n* = 176) we detected 102/176 (58%) samples positive for IgG (IgG seropositivity for index > 1.01), 70/176 (40%) negative (IgG seronegativity for index < 0.9), and 4/176 (2%) with dubious results (IgG dubious results for index between 0.9 and 1.01). Concerning the distribution of data among the maternal blood of the two groups found infected at delivery, in the Late-CoV group (*n* = 58), 34/58 (59%) samples were positive, 23/58 (40%) negative, and 1/58 (2%) showed a dubious result. Comparatively, in the Spread-CoV group (*n* = 8) we found 3/8 (37%) positive and 5/8 (63%) negative samples.

Regarding the cord blood, some samples were not available or were insufficient for the analysis. In the Early-CoV group, 105/165 (64%) samples tested positive (IgG seropositivity for index > 1.01), 49/165 (30%) negative (IgG seronegativity for index < 0.9), and 11/165 (6%) samples showed dubious results. In the Late-CoV group, 24/55 (44%) samples were positive, 28/55 (51%) negative, and 3/55 (5%) were dubious; in the Spread-CoV group, 3/8 (37%) samples were positive and 4/8 (50%) were negative.

**Figure 1:**
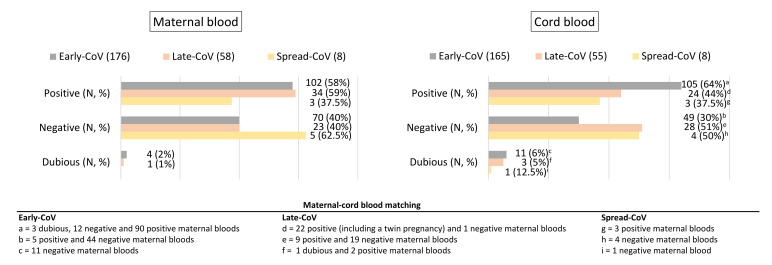
Results of the analysis of SARS-CoV-2-specific IgG in maternal blood and cord blood samples collected within 24 h from delivery. Some cord blood samples were not available or the quantity was not sufficient for the anti-SARS-CoV-2 IgG testing.

According to Yates’s chi-squared test, there was a significant difference in the seroprevalence between the cord blood from the Early-CoV and Late-CoV groups (*p* value= 0.004529) (Figure 1).

### 2.3. Inflammatory Response

**Figure 2:**
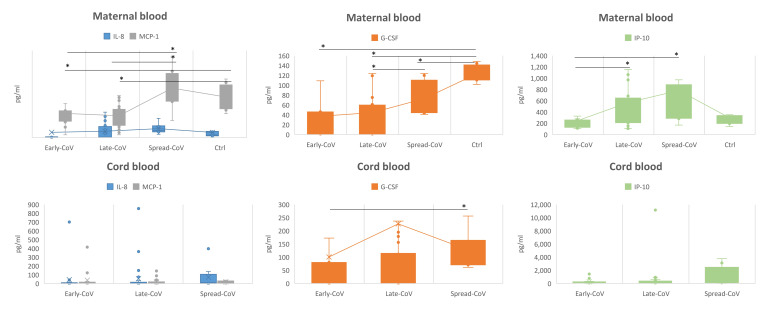
Immune soluble factors. The significantly modulated soluble factors in maternal and cord blood of the analyzed groups. Differences were calculated by means of a Mann–Whitney U test for pairwise comparisons (GraphPad Prism v. 5). * *p* < 0.05.

The profiling of the inflammatory response in maternal sera and cord blood individuated some factors, including IP-10, IL-8, MCP-1, and G-CSF, whose concentration was differently modulated among the CoV groups and the control group (Figure 2).

The concentration of IP-10, IL-8, and MCP-1 increased from the Early-CoV group to the Spread-CoV group and in all the CoV groups with respect to the control group. The growth factor G-CSF, although showing a trend of increase through the CoV groups, showed a lower concentration when compared to the control group.

Concerning the cord blood, a significant result was found only for G-CSF, showing a profile of concentration comparable to that identified in the mothers, with a higher concentration in the Spread-CoV group with respect to the Early-CoV group (Figure 2).

### 2.4. Maternal Microbiome

In a subset of women, vaginal and rectal microbiomes were profiled. Namely, thirty-two women from the Early-CoV group, thirty women from the Late-CoV group, eight women from the Spread-CoV group, and twenty from the control group of uninfected pregnant women.

**Figure 3:**
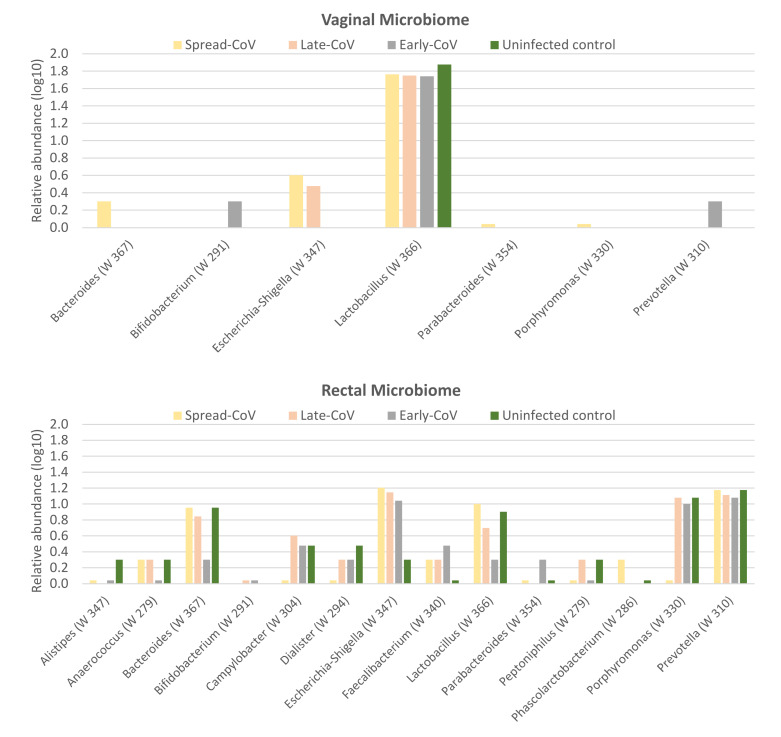
The maternal microbiome. The log-transformed (log10) mean relative abundances of the significantly modulated bacteria, as assessed using the ANCOM test, in rectal and vaginal samples. W values (shown in brackets) indicate the number of times the null hypothesis is rejected by the analysis. The higher the W, the more likely a feature differs statistically.

Figure 3 shows the relative abundances of the significantly modulated bacteria, as assessed in the ANCOM test, in rectal and vaginal samples.

At the time of delivery, the vaginal microbiomes of the CoV groups showed a lower relative abundance of lactobacilli than that observed in the uninfected pregnant women. *Bifidobacterium* was uniquely identified in the vaginal swabs of the Early-CoV group. In the vaginal and rectal microbiomes of the Spread-CoV group, *Bacteroides* and *Escherichia-Shigella* increased compared to the other CoV groups and the control group. In the rectal swabs of the Late-CoV, Early-CoV, and control groups, *Porphyromonas* increased.

**Figure 4:**
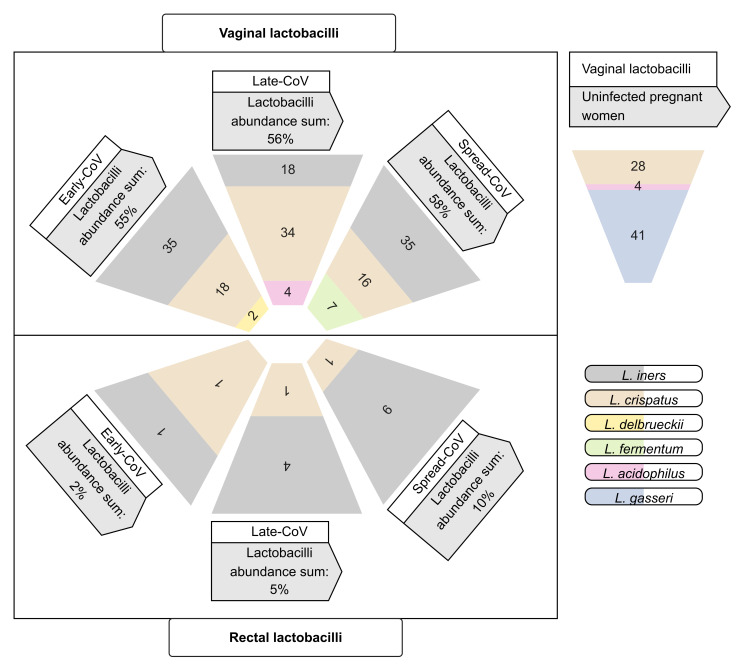
Lactobacilli species. The mean relative abundances of the species of lactobacilli in the vaginal and rectal swabs of the Spread-CoV, Late-CoV, and Early-CoV groups and in the vaginal swabs of uninfected pregnant women at the time of delivery.

Concerning lactobacilli species (Figure 4), *L. iners* was predominant in vaginal swabs from Spread-CoV and Early-CoV groups, while *L. crispatus* was most abundant in the vaginal swabs from the Late-CoV group. *L. fermentum* was uniquely identified in the vaginal swabs from the Spread-CoV group, *L. acidophilus* in the vaginal swabs from Late-CoV group, and *L. delbrueckii* in the vaginal swabs from the Early-CoV group. Compared to the SARS-CoV-2 uninfected pregnant women, *L. crispatus* was less abundant in the Late-CoV and Early-CoV groups.

## 3. Discussion

The data from the present study confirm that the SARS-CoV-2 intrauterine vertical transmission potential is low. In our cohort, only two newborns whose mothers were positive for the virus at the time of delivery acquired the infection (0.83%). One mother was from the Late-CoV group, showing a high viral load in the nasal swab and being infected with the B1.1.1.7 (alpha) SARS-CoV-2 variant. The other one was from the Spread-CoV group, positive in all the analyzed samples for SARS-CoV-2, including the placenta, and being infected with the B.1.617.2 (delta) SARS-CoV-2 variant, which is characterized by a higher intrinsic severity than the alpha variant [23]. In addition, both maternal and cord blood from these two mother–child dyads were negative for anti-SARS-CoV-2 IgG at delivery.

The maternal inflammatory response to the virus emerged as the main possible cause of damage to the fetus, regardless of the in utero infection. Inflammation can affect fetal brain development and inflammatory mediators can pass through the placenta, leading to neuroinflammation [24]. For this reason, in this study, we inspected the immune response to SARS-CoV-2 in pregnant women focusing on the transplacental IgG passage and the dosage of cytokines, chemokines, and growth factors in both maternal serum and umbilical cord blood. The profiling of the vaginal and rectal microbiomes was also evaluated as dysregulation of the immune response could influence the composition of the maternal microbiota [24] and, thus, beneficial neonatal colonization.

Except for the eight women from the Spread-CoV group, SARS-CoV-2 infection was associated in most of the cases with the presence of anti-SARS-CoV-2 IgG at delivery. Concerning the cord blood, a significantly different transplacental passage was observed between Early-CoV and Late-CoV groups. This result could depend on different conditions: it is possible that maternal antibodies from the Late-CoV group were recently produced and were not transferred to the fetus in a timely manner or different kinetics of transplacental antibodies passage occurred in the different trimesters. Indeed, previous data have shown that IgG transfer was proportional to the time elapsed between SARS-CoV-2 infection and delivery, with the transfer increasing as the interval between maternal infection and delivery increased (more than 50 days) [25]. It is a fact that the absence of IgG at birth could leave neonates at risk of infection in the first weeks of life when maternal IgG is supposed to persist in the newborn’s blood [26].

Concerning the immune response, some proinflammatory factors were found to be significantly modulated in maternal blood. In particular, immune proteins associated with a longer duration of illness and disease severity, such as IL-8, IP-10, and MCP-1, were found to be up-regulated compared to controls, reaching the highest concentration in sera of the Spread-CoV group. On the other hand, cord blood did not show a significant variation in the upper mentioned immune factors. This could depend on the fact that the fetus responds to the infection via the secretion of other inflammatory factors and that the maternal immune proteins do not necessarily reach the fetus [4,27].

The growth factor G-CSF, described as one of the players of the COVID-19 cytokine storm, was measured at a lower concentration in the CoV groups if compared to uninfected pregnant women. The same trend was found both in maternal and cord blood [28]. In this regard, G-CSF during pregnancy induces immune tolerance through the switch of the T-cell cytokine secretion profile to Th2 responses [29,30] while MCP-1 is physiologically overexpressed as pregnancy advances, because of the increased trafficking of leukocytes through the uteroplacental unit [31]. Thus, the physiological state of immunomodulation during pregnancy, especially in the last trimester [32], may protect CoV groups from the exacerbation of pro-inflammatory responses due to an active SARS-CoV-2 infection [8,33]. On the other side, G-CSF also has a role in increasing the circulating white blood cells and, in turn, increasing their ability to destroy pathogens [34]. Thus, we could speculate that a decrease in this growth factor at delivery may impact the susceptibility of newborns to neonatal infections, even if we cannot document this result.

Although we cannot elucidate the mechanism of action of SARS-CoV-2 on vaginal and gut microbiomes, our data seem to suggest an unfavorable effect. An association between gut microbiota in SARS-CoV-2-infected pregnant women seemed to take place in our cohort and this could be relevant considering that the gut microbiota not only supports mucosal immunity development but also modulates the systemic immune response in the host [35]. Differences in the rectal microbiome were observed based on the timing of the infection.

In the rectal swabs, the Spread-CoV group showed an increase in proinflammatory bacteria such as *Escherichia-Shigella* which can weaken intestinal bacterial permeability [36]. On the contrary, the increase in *Porphyromonas*, which is a producer of butyric, isobutyric, isovaleric, acetic, and propionic acids, was reported in the Late-CoV, Early-CoV, and control groups [37,38]. The proinflammatory bacteria of the gut microbiomes from the Spread-CoV group, such as *Bacteroides* and *Escherichia-Shigella* [39], were also detected in the vagina. Comparatively, the Early-CoV group showed the presence of *Bifidobacterium*, frequently associated with health-promoting effects in the reproductive and digestive tracts [40].

The major differences in vaginal microbiomes were observed in the distribution of the most-abundant commensal vaginal lactobacilli, which varied in the vaginal swabs of the CoV groups compared to the uninfected pregnant women, which showed the predominance of *L. gasseri* and *L. crispatus*. The Early-CoV and Spread-CoV groups showed the predominance of *L. iners* in the vaginal swabs, usually described in vaginal community types less stable than the other community types and more associated with vaginal dysbiosis [41,42], but two lactobacilli specifically differed between these two groups. Namely, *L. delbrueckii* was identified in the vaginal swabs from the Early-CoV group, recorded as a minor vaginal bacterial species [43] but exhibiting antiviral SARS-CoV-2 properties [44,45]; *L. fermentum*, a probiotic candidate for protecting the intestine against pathogens, was uniquely identified in the vaginal swabs from the Spread-CoV group [46,47]. Concerning the Late-CoV group, *L. crispatus*, widely recognized as an indicator of healthy vaginal microbiota with an antiviral activity towards diverse infectious agents, was identified as the most abundant in the vaginal swabs [48,49,50,51].

In summary, Early-CoV and Spread-CoV groups showed transitional vaginal microbiota but with the Early-CoV group showing probiotic bacteria with anti-SARS-CoV-2 properties such as *Bifidobacterium* and *L. delbrueckii*. This point is relevant as maternal vaginal dysbiosis has been previously described among the causes of premature birth or adverse fetal outcome, including necrotizing enterocolitis (NEC), late-onset sepsis and, successively, food intolerance, with mechanisms potentially involving maternal/fetal microbiota [52,53].

Taken together, our study confirmed that SARS-CoV-2 vertical transmission is a rare event. This is likely due to the maternal antibody’s response and consequent transplacental passage. In our cohort, transplacental passage of anti-SARS-CoV-2 IgG was not observed in pregnant women acquiring infection in the third trimester. In this group, SARS-CoV-2 was correlated with a decrease in G-CSF in both sera and cord blood, in turn possibly decreasing the circulating white blood cells and increasing the risk of infections. Notably, a vaginal and rectal dysbiotic status was observed in women in the Early-CoV group and those with a spread infection. Vaginal and gut dysbiosis have been suggested to have adverse effects on newborns’ health, although in our series the immediate neonatal outcomes were reassuring. Though, this point needs further research.

## 4. Materials and Methods

### 4.1. Study Cohorts and Workflow of the Study

From November 2020 to May 2022, during the COVID-19 pandemic, at the Institute for Maternal and Child Health–IRCCS “Burlo Garofolo”, Trieste, Italy, 242 pregnant women diagnosed with SARS-CoV-2 infection were enrolled in the study.

All pregnancies were singleton, except for one twin pregnancy. Data on the mode of delivery or pregnancy termination, and maternal and neonatal outcomes were subsequently recorded.

Twenty pregnant women (30 ± 5 years) enrolled in a pre-COVID era (hereinafter referred to as uninfected pregnant women), whose vaginal microbiome and dosage of cytokines, chemokines, and growth factors were profiled at the time of delivery, were considered as the control group. All pregnancies were singletons and no pregnancy/delivery complications were reported.

**Figure 5:**
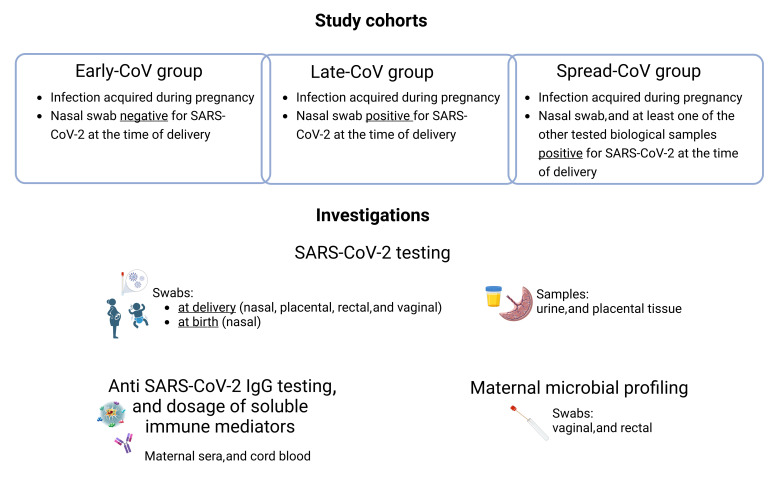
Workflow of the study.

All of the participants were informed and signed written consent. The study was approved by the Institutional Review Board (IRB 03/2020) and all of the experiments were conducted according to the principles stated in the Declaration of Helsinki. The workflow of the study, including type of samples and the type of investigation, is presented in Figure 5.

### 4.2. Specimen Collection

Right after giving birth or at the time of delivery, from 242 pregnant women diagnosed with SARS-CoV-2 infection, nasal, vaginal, rectal, and placental swabs were obtained together with urine, serum, and cord blood. Also, placental biopsies were available. More precisely, nasal swabs were collected using FLOQSwabs (Copan flocked swabs), following the manufacturer’s instructions. A sterile swab with liquid transport medium (cliniswab DS 321/SG, APTACA, Canelli, Italy) was used for vaginal swabs performed by a single gentle 360° rotation of the swab at the vaginal wall, for rectal swabs performed by inserting the cotton swab 1.2 to 2 inches into the rectum, and for placental swabs taken from the amniotic surface after clearing the surface of maternal blood in proximity to the umbilical cord and to two opposite peripheral margins. A sample of fresh placental tissue was obtained and suspended in sterile normal saline. The urine sampling was collected via clean catch into sterile sample cups. Blood specimens for antibody determination were obtained via venipuncture from the mother and funicular puncture sampling with the serum separated from the clot using standard methodology. Before further analysis, samples were stored at −80 °C.

### 4.3. Nucleic Acids Extraction

Viral RNA for SARS-CoV-2 assessment was extracted from nasopharyngeal, vaginal, rectal, and placental swabs and urine samples using the Maxwell RSC Viral Total Nucleic Acid Purification Kit (Promega, Madison, WI, USA) as indicated by the supplier. Before viral RNA extraction, urine samples were centrifuged at 5000× *g* for 20 min, and then the supernatants were discarded, except for 2 mL.

Bacterial DNA for microbiome analysis was extracted from vaginal and rectal swabs using the Maxwell CSC Blood DNA Kit for the Maxwell CSC Instrument (Promega, USA) as indicated by the supplier. All the nucleic acids were stored at −80 °C.

### 4.4. RT–PCR for SARS-CoV-2

The presence of SARS-CoV-2 viral DNA was assessed in nasal, vaginal, rectal, and placental swabs and urine samples using the NeoPlex TM COVID-19 Detection Kit Assay according to the manufacturer’s instructions. This qualitative in vitro assay allows the simultaneous detection of the N and RdRp genes of the SARS-CoV-2 genome, using a real-time reverse transcription polymerase chain reaction (RT-PCR) with a limit of detection of 50 copies of viral RNA (GeneMatrix Inc., Seongnam, Republic of Korea).

### 4.5. COVID-19 Serology

The evaluation of the presence of immune response against SARS-CoV-2 was performed via the automatized StartPlus ELISA system using the SARS-CoV-2 IgG kit (Eurospital Diagnostics, Trieste, Italy) on maternal blood and funicular blood, according to the manufacturer’s instruction. A cut-off value (absorbance of the sample/absorbance of the control) > 1.01 is considered positive for specific SARS-CoV-2 IgG, a cut-off value ranging 0.9–1.01 is considered dubious, and a cut-off value < 0.9 is considered negative.

### 4.6. Analyses of the Inflammatory Response

The inflammatory response was tested on maternal blood and funicular blood using the dosage of 27 analytes including cytokines, chemokines, and growth factors (Bio-Plex Pro Human xMAP Assay, Bio-Rad, Segrate, Italy). The reported limit of detection is 1–20 pg/mL, depending on the target. To normalize the results, cytokine, chemokine, and growth factor concentrations were normalized to total protein in the sample and expressed as pg/mL. The total protein concentrations of samples were determined using the Bradford assay (Sigma-Aldrich, St. Louis, MO, USA). Cytokine median value from a cohort of 20 healthy pregnant women, enrolled independently from this study, was used as a reference.

### 4.7. Microbiome Analysis

To profile the bacterial communities, we sequenced the V3 region of the 16S rRNA gene. Firstly, we used the degenerate primer 27FYM (5′-AGR GTT YGA TYM TGG CTC AG-3′) and the primer U534R, targeting the V1–V3 region (500 bp); subsequently, for the semi-nested PCR targeting the V3 region (200 bp), we used the primers B338F_P1-adaptor (B338F 5′-ACTCCTACGGGAGGCAGC-3′) and U534R_A_barcode (U534R 5′-ATTACCGCGGCTGCTGG-3′). Each PCR reaction (sample) contained a unique IonXpress Barcode Adapter attached to the reverse primer. No-template controls were processed with the clinical samples. The template preparation was performed using the Ion PGM Hi-Q View kit on the Ion OneTouch™ 2 System (Life Technologies, Grand Island, New York, NY, USA) and was sequenced using the Ion PGM Hi-Q View sequencing kit (Life Technologies, New York, NY, USA) with the Ion PGM™ System technology.

The FastQ files were processed using QIIME 2.0, version 2022.2, retaining reads with Q ≥ 20 and a read length 180 bp, after DADA2 denoising. For the taxonomy assignment, Silva v138 was used, with a BLAST+ consensus. Further analysis was carried out on a random subset of 10,000 reads/sample, using a similarity threshold of 97%.

### 4.8. Statistical Analysis

Data analysis was performed using the software GraphPad Prism (v. 5) for immune soluble factors and using QIIME 2 for microbiome results. The difference between groups was tested using the Wilcoxon signed-rank/Mann–Whitney U test. A *p*-value of 0.05 (two-sided) was chosen as the limit of statistical significance. Fisher’s exact test for categorical variables was used, with a significance level of 0.05. To test the differentially modulated bacteria, the ANCOM test was applied.

### 4.9. Compliance with Ethical Standards

All patients provided informed consent for the use of their data and clinical samples for the purposes of the present study. Institutional review board clearance for the scientific use of patient data has been granted to the treating institution by the ethics committee at the IRCCS Burlo Garofolo, Trieste, Italy (IRB 03/2022).

### 4.10. Availability of Data

The data presented in this study are openly available in the SRA NCBI Archive: BioProject ID PRJNA955417.

## Figures and Tables

**Table 1:** Patients’ demographics and pregnancy outcomes.

*Demographics*	Early-CoV	Late-CoV	Spread-CoV	Uninfected Control
Patients	176	58	8	20
Mean age ± SD	33 ± 5.3	31 ± 5.6	35 ± 3.6	30 ± 5
Delivery (mean of weeks + days)	39.1 ± 2.6	39 ± 1.6	37.9 ± 2	39.1 ± 2.2
Vaginal Birth—*n* (%)	148 (84.1)	48 (82.7)	6 (75)	10
C-section—*n* (%)	26 (14.8)	10 (17.3)	2 (25)	10
Spontaneous Abortion	2 (1.1)	0	0	0
SARS-CoV-2 positive newborns (%)	0	3.3	13	0
SARS-CoV-2 negative newborns (%)	100	96.7	87	0
*Delivery complications*				
Pathological CTG ^1^—*n* (%)	11 (6.2)	3 (5.1)	2 (25)	0
Prolonged second stage—*n* (%)	1 (0.5)	3 (5.1)	0	0
Gestosis	2 (1.1)	0	0	0
PROM **	29 (16.5)	6 (10.3)	0	0
*Maternal risk factors*				
Gestational diabetes—*n* (%)	20 (11.4)	10 (17.2)	2 (25)	0
Hypertension—*n* (%)	4 (2.2)	3 (5.1)	0	0
Obesity—*n* (%)	9 (5.1)	2 (3.4)	0	0
COVID-19 pneumonia—*n* (%)	2 (1.1)	4 (6.9)	0	0
*Fetal complications*				
Growth restriction—*n* (%)	11 (6.2)	3 (5.1)	0	0

^1^ cardiotocography; ** premature rupture of membranes.

## Data Availability

The data presented in this study are openly available in SRA NCBI Archive: BioProject ID PRJNA955417.
